# Microbial Communities Associated With Long-Term Tillage and Fertility Treatments in a Corn-Soybean Cropping System

**DOI:** 10.3389/fmicb.2020.01363

**Published:** 2020-06-25

**Authors:** Ali Y. Srour, Hala A. Ammar, Arjun Subedi, Mirian Pimentel, Rachel L. Cook, Jason Bond, Ahmad M. Fakhoury

**Affiliations:** ^1^Department of Plant, Soil and Agricultural Systems, Southern Illinois University, Carbondale, IL, United States; ^2^Department of Botany, Faculty of Science, Zagazig University, Zagazig, Egypt; ^3^Department of Forestry and Environmental Resources, North Carolina State University, Raleigh, NC, United States

**Keywords:** conventional tillage, no-till, NPK, N-only, microbial diversity, ecological guilds, KEGG pathway, fungi

## Abstract

Tillage and fertilization are common practices used to enhance soil fertility and increase yield. Changes in soil edaphic properties associated with different tillage and fertility regimes have been widely examined, yet, the microbially mediated pathways and ecological niches involved in enhancing soil fertility are poorly understood. The effects of long-term conventional tillage and no-till in parallel with three fertility treatments (No fertilization, N-only, and NPK) on soil microbial communities were investigated in a long-term field study that was established in the 1970’s. Here, we used high-throughput sequencing of bacterial, fungal and oomycetes markers, followed by community-level functional and ecological assembly to discern principles governing tillage and fertility practices’ influence on associated soil microbiomes. Both tillage and fertilizer significantly altered microbial community structure, but the tillage effect was more prominent than the fertilizer effect. Tillage significantly affected bacteria, fungi, fusaria, and oomycete beta-diversity, whereas fertilizer only affected bacteria and fungi beta-diversity. In our study different tillage and fertilizer regimes favored specific networks of metabolic pathways and distinct ecological guilds. No-till selected for beneficial microbes that translocate nutrients and resources and protect the host against pathogens. Notably, ecological guilds featuring arbuscular mycorrhizae, mycoparasites, and nematophagous fungi were favored in no-till soils, while fungal saprotrophs and plant pathogens dominated in tilled soils. Conventional till and fertilizer management shifted the communities toward fast growing competitors. Copiotrophic bacteria and fusarium species were favored under conventional tillage and in the presence of fertilizers. The analysis of the metagenomes revealed a higher abundance of predicted pathways associated with energy metabolism, translation, metabolism of cofactors and vitamins, glycan biosynthesis and nucleotide metabolism in no-till. Furthermore, no specific pathways were found to be enriched under the investigated fertilization regimes. Understanding how tillage and fertilizer management shift microbial diversity, structure and ecological niches, such as presented here, can assist with designing farming systems that can maintain high crop yield, while reducing soil erosion and nutrient losses.

## Introduction

Tillage practices and soil fertility management affect soil edaphic factors as well as the biological properties of the soil. In fact, some of these biological properties can be used as valuable indicators of soil quality and health (Carbonetto et al., 2014). A large body of literature reported on the effect of soil tillage and crop residue management on the structure and functions of soil microbial communities (Navarro-Noya et al., 2013; Chavez-Romero et al., 2016; Degrune et al., 2016; Jimenez-Bueno et al., 2016; Srour et al., 2017c). Varied soil physical and chemical factors under different tillage regimes and crop residue management practices, create different habitats for microbes, and result in shifts of the soil microbiome structure (Six et al., 1999; Helgason et al., 2009; Murphy et al., 2016). Furthermore, the soil microbial community is affected by edaphic factors such as temperature, soil moisture, pH, oxygen, nutrients availability (N and P inputs), organic matter, soil texture, and host plants (Ludemann et al., 2000; Kowalchuk et al., 2002; Frey et al., 2008; Lauber et al., 2009; Chau et al., 2011; Brockett et al., 2012; Leff et al., 2015).

No-till (NT) practices with crop residue retention were shown to enrich the soil microbial community. This may be partially attributed to the well documented role that soil microbes play in soil nutrient cycling (He et al., 2007). Moreover, compared to conventional tillage (CT), NT systems were found to be associated with higher bacterial and fungal species diversity (Helgason et al., 2009, 2010). With NT, fungal and bacterial species generally categorized as decomposers are usually found to be predominant in soils (Spedding et al., 2004; Nicolardot et al., 2007). Similarly, arbuscular mycorrhizal fungi (AMF), are usually more abundant under conservation tillage (van der Heijden et al., 1998; Alguacil et al., 2008; Sale et al., 2015). AMF are often considered to be natural biofertilizers supplying the host with water and nutrients and protecting it from root pathogens in exchange for photosynthetic products. Moreover, soil microbial biomass and enzymatic activities related to soil nutrients cycling were reported to improve with conservation tillage practices (Salinas-Garcia et al., 2001; Zhang et al., 2013; Murphy et al., 2016). The concentration and the availability of nutrients (e.g., organic C, N, P, and K) in the soil, whether coming from mineralization or from direct fertilization, have been considered to provide a good assessment of soil quality and fertility (Vogeler et al., 2009; Cao et al., 2011). Soil organic carbon and total N are the most favorable indices of soil fertility (Puget et al., 2000; Deen and Kataki, 2003; Polyakov and Lal, 2004; Olson et al., 2005). Given the close relationship that exists between soil organic carbon, total nitrogen and microbial metabolic activities, the dynamics of C and N accumulation in soils not only improve soil physical parameters, preserve soil moisture, and reduce erosion, but also drive microbial activity and nutrient cycling (Manna et al., 2007).

Several studies have shown that the long-term continued application of inorganic fertilizers may result in an adverse effect on soil quality and productivity (Kumar and Yadav, 2001; Zhou et al., 2016). It has been reported that the long-term application of fertilizers may not necessarily improve the levels of nutrients in the soil. In fact, it has been reported that soil organic carbon and total nitrogen levels may decrease under those conditions (Shen et al., 2004; Su et al., 2006). Nevertheless, in other studies, the levels of soil organic carbon, total nitrogen and other nutrients were found to be higher following long-term fertilizers regimes (Whitbread et al., 2003; Huang et al., 2010). Therefore, there is still a lot to be learned regarding the effect of various tillage and fertilization treatments on soil fertility, and associated biological properties. In addition to the chemical and physical characteristics of the soil, The soil microbiome has been gaining increasing attention lately as an important soil quality assessment measure (Schloter et al., 2003, 2018).

A tillage by fertilizer field experiment has been in place at Southern Illinois University Belleville Research Center (BRC) since 1970 on a somewhat poorly drained soil. The effects of tillage and fertilizer on crop yield and soil physical and chemical properties have been studied extensively (Cook and Trlica, 2016), however we know far less about impacts on soil biology. In no-till with application of NPK fertilizer, crop yield and profits were found to be comparable with other tillage systems in corn-soybean production (Trlica et al., 2017). No-till with NPK increased soil organic matter (OM) to 27.6 g kg–1 with the greatest increase in the surface soil layer (0–5 cm) compared to conventional tillage. In NPK treatment, available P and K declined significantly from 1990 to 2013 to near recommended levels in both no-till and conventional till. No-till treatments showed stratification of P and K, but it had no effect on yield. No excessive pH stratification was observed between tillage treatments, and soil pH values were similar among fertilizer treatments in 2013 (Cook and Trlica, 2016).

We hypothesized that:

(1) Microbial communities under different tillage and fertility treatments will vary in their richness, diversity, and niche-based processes.
(2) No-till, and NPK treatments will have higher microbial diversity and richness due to increase in organic matter.
(3) Stratification of nutrients and minor soil disturbances in no-till management will favor symbiotrophs, whereas nutrients homogenization in conventional till will promote saprotrophs.
(4) Plant pathogens will be favored in non-tilled plots due to absence of soil disturbance.

Using next-generation sequencing of various DNA markers representing bacteria, fungi, oomycetes and fusaria communities, and analyses of associated metabolic pathways and ecological services involved, we compared soil microbial communities in the presence or absence of tillage (CT and NT), in conjunction with external fertilizers input (no fertilizer, N-only, NPK) in a field scale experiment at Southern Illinois established more than 45 years ago.

## Materials and Methods

### Field Site Description

The field site for the study is located at the Southern Illinois University Belleville Research Center (BRC) near Belleville, IL, United States (38.519179° N, 89.843248° W). The site was established in 1970 on a somewhat-poorly drained Bethalto silt loam (fine-silty, mixed, superactive, mesic Udollic Endoaqualf) soil. The soil had 15 g Kg-1 organic matter in the surface 15 cm, 10 cmol kg^–1^ of CEC, pH 6.1, 34 kg ha^–1^ of available P, and 292 kg ha^–1^ of exchangeable K at the initiation of the study in 1970 (Kapusta et al., 1996). The field was arranged in a split-plot design with “Tillage” as the main plot and “Fertilizer” as the subplot.

From 1970 to 1990, corn was planted continuously. Since the beginning of 1991, the plots were shifted to a corn-soybean rotation (Kapusta et al., 1996). The site consisted of two tillage treatments: (i) moldboard till; and (ii) no-till with no soil disturbance. Fertilizer treatments comprised: (i) no fertilizer (Control); (ii) nitrogen only (N-only); and (iii) NPK only (NPK) randomized within each tillage strip (Kapusta et al., 1996; Cook and Trlica, 2016). Fertilizer treatments were only applied in years during which corn was planted. Specific fertilizer application rates are provided in Supplementary Table S1. Additional field history and treatment details can be found in Cook and Trlica (2016).

In this study, we had six treatment combinations (CT and NT) × (Control, N-only, and NPK). Three replications per treatment combination (fertilizer × tillage) were selected for microbial analysis, resulting in a total of 18 soil samples.

### Soil Sampling

Soil samples were collected in the fall of 2015 at soybean growth stages R4-R6. Soil samples were taken at a depth of up to 20 cm, close to the roots, using a stainless-steel probe. Three replicates, each consisting of five cores, were taken from each treatment. Soil cores from each replicate were combined and mixed (resulting in three samples per treatment). Soils were homogenized, sieved to remove plant debris, and stored at −80°C in 50-ml plastic tubes until further analysis.

### High Throughput Sequencing

Total DNA was extracted using a MOBIO PowerLyzer PowerSoil DNA Kit (Qiagen, Carlsbad, CA, United States) with a minor modification in the incubation step as previously described (Srour et al., 2017a). For each of the 18 soil samples, three technical replicate extractions were pooled together. DNA concentration and quality (A_260_/A_280_) was assessed using a Nanophotometer P-30 (Implen, Munich, Germany), and by agarose gel electrophoresis (Sub-Cell GT, Bio-Rad, Hercules, CA, United States).

The V4 region of the 16S rRNA gene was amplified using the modified primers 515F and 806R (Apprill et al., 2015; Parada et al., 2016) which are suitable to target both bacterial and non-eukaryotic archaeal taxa. For fungi, we used ITS3 and ITS4 primers to amplify the internal transcribed spacer region 2 (ITS2) (White et al., 1990). To detect Oomycetes, we used ITS6 and ITS7 primers targeting the ITS1 region (Cooke et al., 2000). For *Fusarium* species, Alfie1 and Alfie2 primers were also used to target a partial region of the translation elongation factor-1 alpha (EF-1 alpha) gene (Yergeau et al., 2005).

For each target region, the forward and reverse primers were tagged with a Fluidigm-specific primer pad CS1: 5′-ACACTGACGACATGGTTCTACA-3′ and CS2: 5′-TACGGTAGCAGAGACTTGGTCT-3′, respectively. All primer pairs were purchased from IDT (Coralville, IA, United States). PCR was conducted according to Fluidigm Access Array protocols (Fluidigm, CA), a detailed protocol is provided in Supplementary File. Harvested Fluidigm products were then transferred to a new 96 well plate and diluted 1:100 in water.1 μl of the diluted product was used for a second round of amplification with Illumina linkers and barcodes. The resulting Fluidigm pools were submitted to the DNA Services Laboratory at the W. M. Keck Center at the University of Illinois at Urbana–Champaign. The final pools were quantitated using Qubit (Life Technologies, Grand Island, NY, United States), and the average size determined on an Agilent bioanalyzer HS DNA chip (Agilent Technologies, Wilmington, DE, United States) and diluted to 5 nM final concentration. The 5 nM dilutions were further quantitated by qPCR on a BioRad CFX Connect Real-Time System (Bio-Rad Laboratories, Inc. CA) and pooled evenly. The final denatured library pool was spiked with 20% non-indexed PhiX control library provided by Illumina and loaded onto the MiSeq V2 flowcell at a concentration of 7 pM for cluster formation and sequencing. The libraries were sequenced from both ends of the molecules to a total read length of 250 nt from each end.

The run generated .bcl files were converted into demultiplexed compressed fastq files using bcl2fastq 2.17.1.14 (Illumina, CA). A secondary pipeline decompressed the fastq files, generated plots with quality scores using FastX Tool Kit, and generated a report with the number of reads per sample/library. The .bcl files were then sorted by initial PCR-specific primer. Both primer-sorted and demultiplexed fastq files were .tgz compressed.

The 16S rRNA, ITS1, ITS2 and EF1 alpha gene sequences were submitted to the NCBI Sequence Read Archive with the Accession Number SRP242303.

### Bioinformatics Analysis

Microbiome sequencing data were analyzed with the Quantitative Insights Into Microbial Ecology (QIIME 2) software package (Caporaso et al., 2010). Raw sequences were demultiplexed and quality filtered using the q2-demux plugin followed by denoising with the q2-dada2 plugin (Callahan et al., 2016) to produce amplicon sequence variants (ASVs); i.e., to produce 100% Operational Taxonomic Units (OTUs) representing the entirety of unique sequences observed in the dataset. All ASVs were aligned with q2-alignment and used to construct a phylogeny with q2-phylogeny. Alpha-diversity metrics (observed OTUs and Shannon index) were estimated after samples were rarefied to 1200 sequences per sample (to retain as many samples as possible). Samples with fewer sequences than the rarefaction depth were excluded from downstream diversity analyses. Closed reference OTU picking was done against the Greengenes 13.8 database for bacterial 16SrRNA, the Unite 7.2 database for fungal ITS, and custom databases for oomycetes ITS and Fusaria EF1α. The term “OTU level” used here refers to individuals assigned to their lowest common ancestor across multiple taxonomic ranks.

Metagenomic inference and functional composition of bacterial 16S data was performed using Phylogenetic Investigation of Communities by Reconstruction of Unobserved states (PICRUSt) (Langille et al., 2013). The accuracy of metagenome predictions was measured using the weighted Nearest Sequenced Taxon Index (NSTI) scores which serve as indicators for the availability of reference genomes closely related to the microorganisms in our samples. Pathway analysis was performed using Kyoto Encyclopedia of Genes and Genomes (KEGG) pathways (Kanehisa and Goto, 2000). Functional annotation of fungal ITS was conducted using a curated FUNGuild v1.1 database^1^. FUNGuild was used to assign ecological functions or trophic modes to each fungal OTU (Nguyen et al., 2016). Computational work used BigDawg High Performance Computing Cluster (HPCC) located at Southern Illinois University, Carbondale, IL, United States (Langin, 2019).

### Statistical Analysis

For the statistical analyses we tested the effect of the following factors: *tillage regime* with CT and NT, and *Fertilizer management* with NPK, N-only and Control (No fertilizer) on soil microbial communities. Shannon index of diversity (H) and Observed OTUs within the tillage and fertilizer treatments were analyzed using generalized linear mixed model analysis with proc glimmix in SAS 9.4 (SAS Institute Inc., Cary, NC, United States). The model estimated the fixed effects of tillage, fertilizer, and interaction effects of tillage by fertilizer and random effects of replicates. Means were separated using Tukey’s honest significant difference (HSD) at alpha level of 0.05.

A crossed design was similarly used to test the effects of fertilizer and tillage factors on soil microbial assemblages. Differences in β-diversity were examined using the Bray–Curtis similarity calculated from normalized and log (x + 1) transformed OTU abundances. The significance of the experimental factors was tested using multivariate permutational analysis of variance (PERMANOVA) as implemented in Primer7+ with 9,999 permutations (Primer-e, New Zealand). The major variance components of bacterial, fungal, oomycetes and fusaria β-diversity were visualized using Principal Coordinate Analysis (PCoA) and Canonical Analysis of Principal Coordinates (CAP) as described in Srour et al. (2017b). Differentially abundant OTUs were detected using analysis of percent similarity (SIMPER) as implemented in Primer7+.

Mean proportions of fungal ecological guilds and bacterial enriched KEGG pathways were compared and visualized using Statistical Analysis of Metagenomic Profiles (STAMP) (Parks et al., 2014) to determine functions and modes that had significant differences between treatments (NT, CT, Control, N-only, and NPK). A supervised random forest model was used to identify discriminatory taxa as implemented in the randomForest (version 4.6-14) package in R^2^. Random forest is an ensemble learning method well adapted to perform classification and regression analyses by aggregating many predictions to reduce the variance and improve robustness of outputs (Breiman, 2001). Random forest classification starts by constructing multiple decision trees using the bagging approach where each tree is constructed independently from a bootstrap sample of the entire dataset. To classify an unknown sample, new data are mapped along the multiple decision trees built to train the model making the voting tree. To evaluate the accuracy of classifications, an error rate of the global prediction, so-called “out-of-bag error rate,” is estimated. For that, during tree growth, the bootstrap sample repetition omits about one-third of the training samples, constituting the out-of-bag samples. Considered as new unknown samples, out-of-bag samples are classified among the different sources using the majority vote. For each model, a misclassification rate, i.e., out-of-bag error rate, is assessed by aggregating the cross-validation results between the predictions and the true sources. Mean decrease in accuracy for a variable (taxa) denotes the normalized difference in the classification accuracy for the out-of-bag data when that variable is included versus when data for that variable are randomly permuted.

## Results

### Composition of Bacterial, Fungal, Fusaria and Oomycetes Communities

Illumina MiSeq runs yielded a total of 326,005 bacterial, 234,141 fungal, 315,411 oomycetes, and 271,331 fusaria raw reads, respectively. After denoising, quality filtering and trimming, a total of 234,174 bacterial 16S, 155,580 fungal ITS, 224,805 oomycetes ITS, and 158,673 EF1-α fusaria sequences were obtained from 18 soil samples. Denoised high quality sequences were binned into 4,994 bacterial, 858 fungal, 847 oomycetes, and 868 fusaria OTUs. Bacterial and archaeal communities were dominated by the phyla Proteobacteria (22.77%), Acidobacteria (17.43%), Actinobacteria (13.18%), Verrucomicrobia (13.16%), Crenarchaeota (9.31%), Chloroflexi (6.26%), Planctomycetes (4.11%), Nitrospirae (3.63%), Gemmatimonadetes (2.79%), Bacteroidetes (2.51%), and Firmicutes (1.08%).

Fungal communities were dominated by the phyla Ascomycota (60.01%), Basidiomycota (13.91%), Mortierellomycota (8.87%), Glomeromycota (1.40%), and Rozellomycota (1.01%). Oomycetes communities were dominated by Peronosporales (12.31%), Pythiales (8.59%) and Lagenidiales (2.13%). Fusaria species were dominated by the *Fusarium oxysporum species complex* (25.4%), and the *Fusarium solani species complex* (14.3%), followed by the *Fusarium incarnatum-equiseti species complex* (6.03%).

### Alpha Diversity of Different Tillage and Fertilizer Regimes

Rarefaction curves tended to an asymptote indicating that the sequencing depth of the majority of samples was sufficient for an exhaustive exploration of the bacterial, fungal, oomycetes and fusaria communities present in each sample (Supplementary Figure S1). Observed OTUs and Shannon’s diversity indices (H) were calculated at the OTU level assigned to the lowest possible taxonomic rank. The OTU level uses assigned taxonomy across multiple ranks (kingdom to species).

Of the whole microbial community tested, just bacteria and fusaria alpha diversities were significantly affected by tillage regime, whereas fertilizer treatment only affected fusaria alpha diversity. In addition, there was no significant interaction effect of tillage and fertilizer on the Shannon index of diversity (H) and observed OTUs within the bacterial, fungal, and oomycete communities. Tillage treatment showed a significant effect on the bacterial community with higher diversity (H) in CT compared to NT (*P* = 0.037, Supplementary Table S2), whereas fertilizer had no effect. Tillage treatment also affected the fusaria communities with higher number of OTUs in CT compared to NT (*P* = 0.026, Supplementary Table S2). In addition, an interaction of tillage and fertilizer treatment had significant effect on fusaria alpha diversity (*P* = 0.012, Supplementary Table S2). The NT-Control treatment combination had the lowest Shannon index (H’), whereas the NT-NPK, CT-NPK, and CT-N-only had significantly higher H’ than NT-Control. The combinations NT-N-only and CT-Control had intermediate H’ values (Supplementary Table S2).

### Effect of Long-Term Tillage on Beta Diversity

**FIGURE 1:**
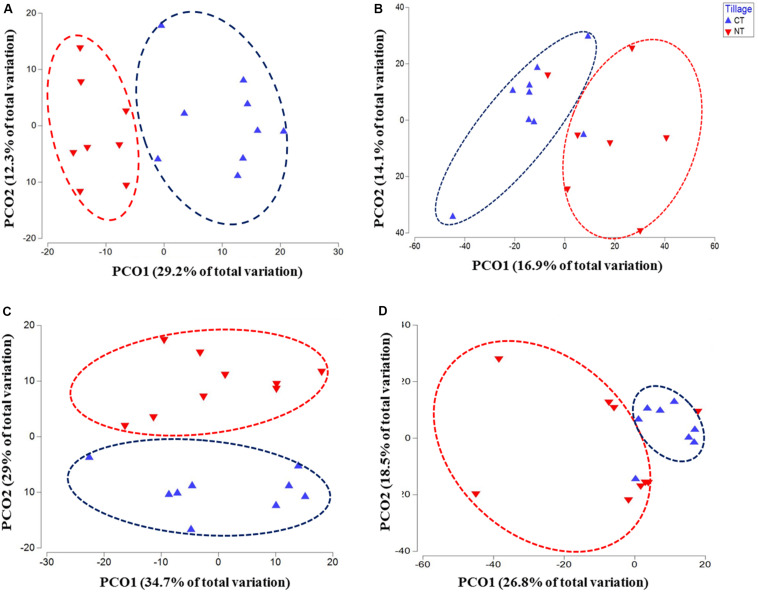
Principal coordinates ordination (PCoA) of microbial communities grouped by tillage regimes based on Bray–Curtis distance. **(A)** Bacteria, **(B)** Fungi, **(C)** Oomycetes, and **(D)** Fusaria; CT, conventional tillage in blue; NT, no-till in red.

The changes in microbial community beta diversity under different tillage and fertility treatments were assessed by calculating the Bray-Curtis dissimilarity among the samples. PERMANOVA test showed no significant interaction between tillage and fertilizer treatments in the microbial communities, however, the main factors tillage and fertilizers affected the microbial community differently (Table 1). In this study, tillage emerged as the main factor driving beta-diversity in the detected soil microbial communities, including fusaria, bacteria, oomycetes and fungi (*P* < 0.05, Table 1). Microbial community structure differed significantly between CT and NT samples as shown after using a PERMANOVA test. Further, dissimilarity shifts in bacterial and fungal beta diversities were evident in the PCoA ordination, with soil samples clustering by tillage factor across the first axis (Figures 1A,B).

**TABLE 1:** PERMANOVA analysis based on Bray–Curtis dissimilarities illustrates factors (tillage and fertilizer) affecting each microbial community structure.

	*Bacteria*						Fungi				
Main test	df	SS	MS	Pseudo-F	P(perm)	Unique perms	SS	MS	Pseudo-F	P(perm)	Unique perms
Tillage	1	1446.3	1446.3	5.0267	**0.0009**	9916	4934.2	4934.2	1.9763	**0.0097**	9893
Fertilizer	2	849.35	424.67	1.476	**0.0413**	9874	7112	3556	1.4243	**0.0449**	9870
Tillage x Fertilizer	2	563.97	281.99	0.98008	0.5285	9862	5542.5	2771.2	1.1099	0.3083	9868
Residual	11	3164.9	287.72				24967	2496.7			
Total	16	6090.7					42325				

	**Oomycetes**						**Fusaria**				
**Main test**	**df**	**SS**	**MS**	**Pseudo-F**	**P(perm)**	**Unique perms**	**SS**	**MS**	**Pseudo-F**	**P(perm)**	**Unique perms**

Tillage	1	1736.2	1736.2	5.0636	**0.0012**	9947	2061.2	2061.2	1.8296	**0.0467**	9928
Fertilizer	2	275.93	137.97	0.40237	0.9266	9941	2130.9	1065.4	0.94571	0.5575	9904
Tillage × Fertilizer	2	770.81	385.4	1.124	0.3445	9948	1883	941.51	0.83572	0.6885	9915
Residual	11	4114.6	342.88				12392	1126.6			
Total	16	6897.6					18468				

*Bold numbers indicate significance (*P* < 0.05). *P-*values are based on 9999 permutations.*

Similar results were observed in fusaria and oomycetes communities, where tillage was the main factor driving beta-diversity. Using an unconstrained PCoA ordination, Fusaria and oomycetes communities were clustered mainly by tillage factor across the first and second axis respectively (Figures 1C,D). These findings suggest that tillage can exert a strong effect in structuring microbial communities when applied over a long-term period.

### Effect of the Long-Term Use of Fertilizers on Beta Diversity

We observed significant differences between fertilizer treatments in bacterial, and fungal communities based on Bray–Curtis dissimilarity. Bacterial and fungal community structure differed significantly between different fertility groups as revealed by using a PERMANOVA test (Table 1). The “Fertilizer” factor had a significant effect on those community structures, with significant differences between the “Control,” “N-only,” and “NPK” fertility groups (*P* < 0.05; Table 1). However, fusaria and oomycetes communities seemed to be less impacted by fertility treatment.

**FIGURE 2:**
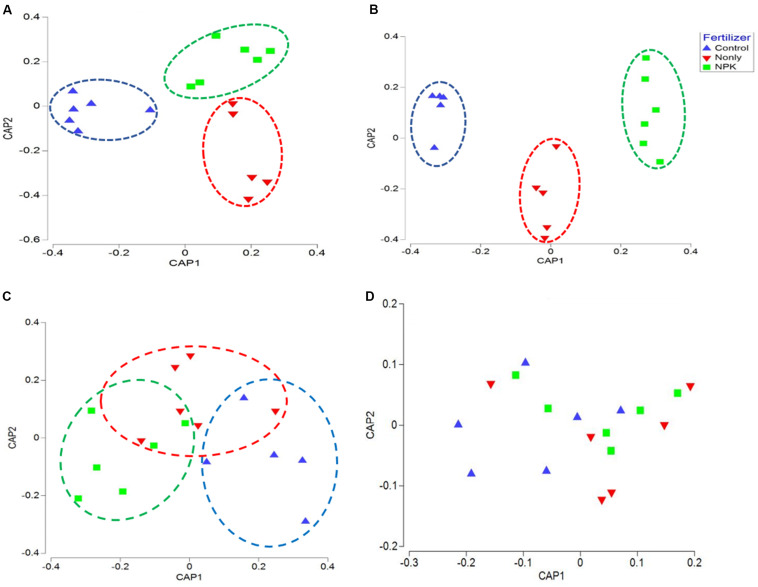
Canonical analysis of principal coordinates (CAP) ordination of microbial communities grouped by fertilizer treatments (Control, N-only, and NPK) based on Bray–Curtis distance. **(A)** Bacteria, **(B)** Fungi, **(C)** Fusaria, and **(D)** Oomycetes; Control, no fertilizaton in blue, N-only in red, and NPK in green.

The correlation between the composition of microbial taxa and the “fertilizer” factor was further confirmed using canonical analysis of principal coordinates (CAP). CAP plots of biome taxa showed a distinct clustering among samples associated with the same fertility treatment: NPK fertility groups were clustered closer together, separately from the Control groups, across either first or second axes. Likewise, “N-only” fertility groups were found clustered separately from Control and NPK groups in bacterial, and fungal CAP analyses (Figures 2A,B). Although fertility was not found to be a significant factor impacting Fusaria (Table 1), there was clear clustering of fusaria communities by fertility treatment in the CAP plot. In particular, the Control and NPK groups were separately clustered away from each other (Figure 2C). In contrast, there was no distinct clustering of oomycetes communities by fertility treatment, suggesting relatively less variability in oomycetes community structure under different fertility treatments (Figure 2D).

### Niche Partitioning in Fungi Across Tillage and Fertilizer Treatments

**FIGURE 3:**
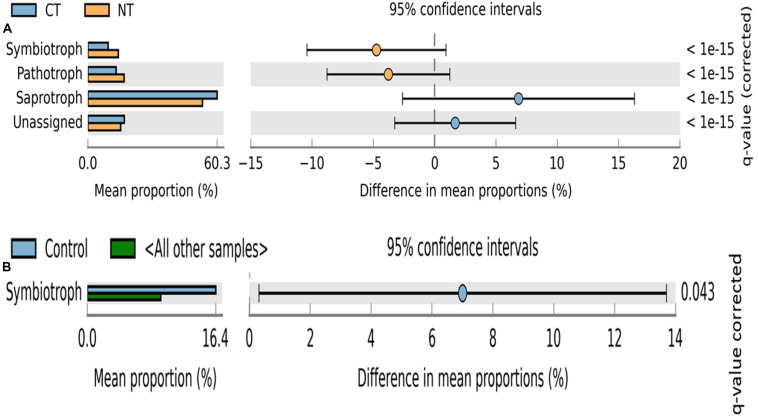
Extended error bar plot identifying significant trophic modes assignments between mean proportions of fungal OTUs. **(A)** in tillage treatments; NT, no-till (orange) vs. CT, conventional till (blue) samples, and **(B)** in fertilizer treatments; Control (blue) vs. other samples (N-only and NPK).

**FIGURE 4:**
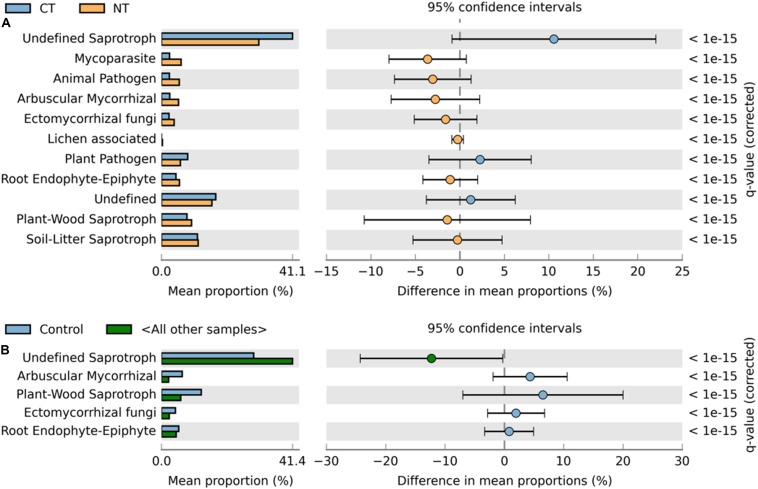
Extended error bar plot identifying significant functional guilds assignments between mean proportions of fungal OTUs. **(A)** In tillage treatments; NT (orange) vs. CT (blue) samples, and **(B)** in fertilizer treatments; Control (blue) vs. other samples (N-only and NPK, in gray). Corrected *q*-values are shown at right.

Fungal OTUs were classified into three main trophic modes (pathotrophs, symbiotrophs, and saprotrophs) and ten subcategories of functional guilds (animal pathogen, plant pathogen, mycoparasite, arbuscular mycorrhizal fungi, ectomycorrhizal fungi, root endophyte, lichenized fungi, undefined saprotroph, soil-litter saprotroph, and plant-wood saprotroph) (Nguyen et al., 2016). On average, 80% of fungal OTUs were successfully assigned to a functional guild, and less than 20% of OTUs were left unassigned or undefined (Figures 3, 4). Trophic modes’ relative abundances differed among tillage and fertility treatments. Symbiotrophs and pathotrophs had greater mean abundances in NT compared to CT (Figure 3A). With regard to fertility treatments, symbiotrophs dominated in Control (no fertilizer) and constituted the only trophic mode that differed significantly among the three considered trophic modes (Figure 3B).

Similarly, relative abundance of major functional guilds varied among tillage and fertility treatments. The groups mycoparasites, arbuscular mycorrhizae, animal pathogens, soil-litter and plant-wood saprotrophs showed greater abundance in NT than in CT. Whereas, in CT, undefined saprotrophs and plant pathogens dominated (Figure 4A).

Among fertility treatments, members of arbuscular mycorrhizae, and plant-wood saprotrophs were more abundant in the Control than in N-only and in NPK samples. Whereas, groups of undefined saprotrophs and soil-litter saprotrophs were more abundant in N-only and NPK compared to the Control (Figure 4B).

### Bacterial KEGG Pathways Enriched in Tillage and Fertilizer Treatments

**FIGURE 5:**
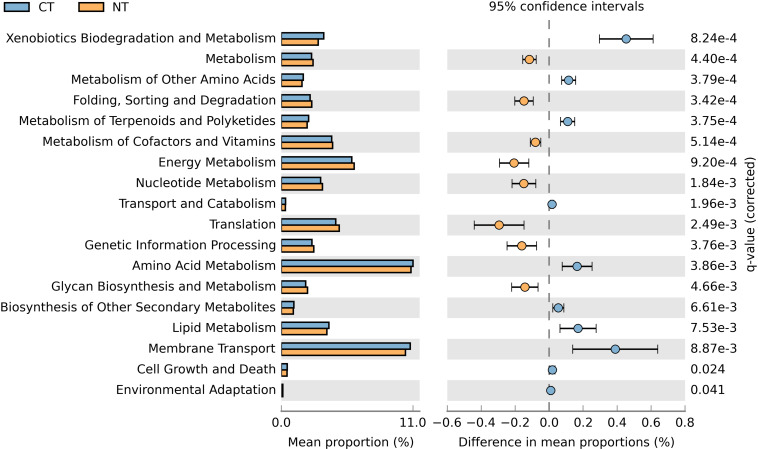
Prediction of changed KEGG pathways using PICRUSt analysis in response to treatments; A total of 18 KEGG pathways were significantly changed between CT (blue) and NT (orange).

In order to obtain a robust bacterial functional profile, bacterial 16SrRNA sequences datasets were used in PICRUSt to predict a KEGG-based functional profile (Langille et al., 2013). Metagenome prediction from 16S rRNA sequences was evaluated with a high NSTI value of 0.23 ± 0.02, which is expected for soil samples as reported in previous studies (Zarraonaindia et al., 2015; Lopes et al., 2016). A total of 4,800 protein-coding genes were predicted using the Clusters of Orthologs Groups (COGs) (Tatusov et al., 1997), of which 643 were differentially represented in CT or NT (*P* < 0.05) in accordance to their frequencies; 329 were significantly more abundant in NT regime, and 314 were significantly higher in CT regime. The differentially abundant protein-coding genes were mapped to 41 KEGG pathways using the KEGG level 2 GO. A Welch’s *t*-test indicated that NT and CT were enriched in 8 and 10 functional categories (KEGG level 2), respectively (*P* < 0.05). More specifically, predicted genes more abundant in the NT regime were mainly associated with metabolism, energy metabolism, glycan biosynthesis and metabolism, genetic information procession, translation, metabolism of cofactors and vitamins, nucleotide metabolism and ‘folding, sorting and degradation’ (Figure 5). On the other hand, genes related to xenobiotics biodegradation and metabolism, amino acid metabolism, metabolism of other amino acids, lipid metabolism, transport and catabolism, metabolism of terpenoids and polyketides, biosynthesis of other secondary metabolites, cell growth and death, and environmental adaptation were more prevalent in the CT regime (Figure 5).

When fertility treatments were investigated, none of the functional categories showed significantly altered enrichment patterns between Control (No fertilizer) and other fertility treatments (N-only and NPK), or between N-only and NPK. The absence of observed enriched KEGG pathways differing between fertility groups may be due to the limited sample size used in this study and the high variability among samples from the same fertility group. As reported by Parks et al. (2014), functional profiles often display large variability. Under these circumstances, the sample size may affect the statistical power of a Welch *t*-test with Benjamini–Hochberg FDR correction, thus reducing the ability of these tests to ascertain a small effect size as statistically significant.

### Differential Responses of Bacterial Groups at High and Low Taxonomical Ranks to Tillage and Fertilizer Treatments

Tillage regime resulted in significant divergence in bacterial relative abundance at the high taxonomical ranks (Phylum and Class) as well as at the low OTU level. The bacterial phyla Chloroflexi, Gemmatimonadetes, Actinobacteria, Armatimonadetes, Chlorobi, Firmicutes, and Proteobacteria were more abundant under CT, whereas Nitrospirae, Verrucomicrobia, TM6 (recently named Dependentiae), WS3 (also called Latescibacteria), Crenarchaeota, Cyanobacteria, and Parvarcheaota increased in relative abundance under NT (Supplementary Figure S2A). Similarly, there were shifts in relative abundance at the class level; major classes of bacteria, including Gemmatimonadetes, Alphaproteobacteria, Thermoleophilia, Ellin6529 (Chloroflexi), and Solibacteres increased in relative abundance under CT, whereas the relative abundance of Nitrospira, Acidobacteria_6, and Spartobacteria increased under NT (Supplementary Figure S2B).

Differential abundance to tillage and fertility regimes was also determined at the OTU level. A total of 105 (30%) bacterial OTUs were dissimilar between CT and NT as revealed by SIMPER analysis. Among those, 74 bacterial OTUs responded positively to CT, and 31 OTUs responded positively to NT. The distribution of these OTUs across tillage regimes is shown in Supplementary Table S2. Top OTUs belonging to the taxonomic groups Kaistobacter, Oxalobacteraceae, Gaiellaceae, Ellin6529, Bacillus, and Solirubrobacterales were well represented under the CT regime. Whereas, members of SCA1170, DA101 (Verrucomicrobia), Chitinophagaceae, 0319-6A21 (Nitrospirales), and RB40 (an uncharacterized order of Acidobacteria) showed a largely uniform increase under NT regime.

No variability was observed with the different “Fertility effects” among the higher-order taxonomic groups of bacteria; none of the bacterial phyla or classes were significantly affected by the different treatments. However, many bacterial OTUs, identified at lower taxonomic ranks, had significantly different abundance levels with the different fertility treatments (Supplementary Tables S4, S5). For instance, Ellin329, Koribacteraceae, Kaistobacter, Candidatus Nitrosphaera, Chitinophagaceae, WMSP1 (uncharacterized order of Chloroflexi), Patulibacteraceae, and Frankiaceae co-occurred and were relatively more frequent in N-only and NPK samples compared to Control samples. On the other hand, several taxa belonging to Acidobacteria, Rhizobiales, Cupriavidus, and Pirellulacaea were relatively more abundant in the Control. Based on the existing literature, the ecological relevance and potential lifestyles of the most salient tillage- and fertility responsive taxa will be discussed in the next section.

### Response of Fungal Taxa to Tillage and Fertilizer Treatments

**FIGURE 6:**
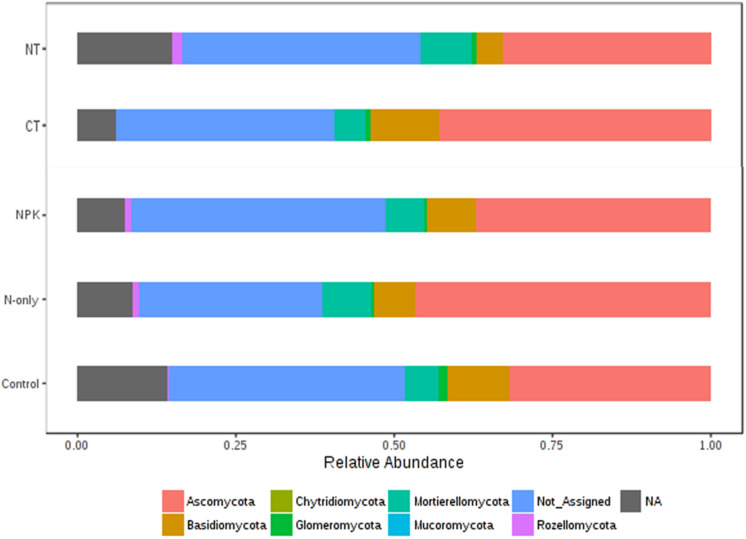
Changes in the relative abundance of fungal phyla under different tillage and fertilizer treatments; CT (conventional till), NT (no-till), Control (no fertilizer), N-only, and NPK.

Tillage effect on divergences in the relative abundance of the predominant fungal phyla ascomycota, basidiomycota, and mucoromycota was observed. These taxa were more abundant under CT (*P* < 0.01). On the other hand, mortierellomycota, glomeromycota, chytridiomycota, and rozellomycota were more abundant in NT (*P* < 0.01; Figure 6). Fertilizer effects on higher-order taxonomic groups of fungi showed also high dissimilarity, mainly between the Control and the other fertilizer groups; NPK and N-only showed some degree of variability but with no statistical significance (Figure 6). The majority of fungal phyla listed above were found to be more abundant in the fertilizer groups N-only and NPK (*P* < 0.01) compared to the Control, with the exception of basidiomycota and glomeromycota, which showed more abundance in the Control group (Figure 6).

**FIGURE 7:**
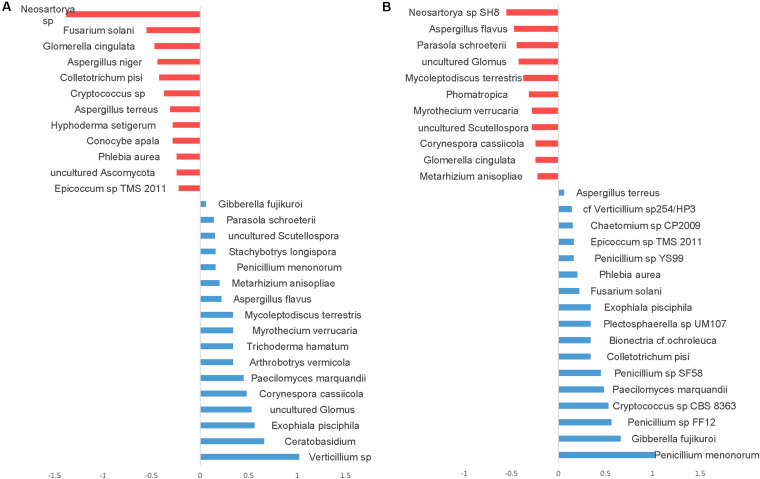
Similarity percentage analysis (SIMPER) of fungal OTUs affected by tillage and fertilizer treatments. **(A)** Relative abundance of fungal taxa influenced by tillage treatment. Mean taxa abundances of NT were subtracted from CT. Taxa that are more abundant in NT are right of the center axis (blue), while taxa more abundant in CT (red) are left of the center axis; **(B)** fungal taxa influenced by fertilizer treatments; NPK and N-only in blue, Control in red.

Differential abundance with tillage and fertilizer regimes was also determined at the OTU level. A total of 37 (24.7%) fungal OTUs were dissimilar between CT and NT when SIMPER analysis was used. Among those, 17 fungal OTUs responded positively to CT, whereas 20 fungal OTUs responded positively to NT. Top OTUs dissimilar across tillage regimes are shown in Figure 7A. Taxa most associated with NT included *Verticillium* sp., *Corynespora cassiicola*, arbuscular mycorrhizal fungi (*Glomus* sp., Scutellospora), saprotrophs (Ceratobasidium, *Exophiala pisciphila*, *Stachybotrys longispora*), and several nematophagous fungi including *Paecilomyces marquandii*, *Arthrobotrys vermicola*, *Myrothecium verrucaria*, *Mycoleptodiscus terrestris*, and *Metarhizium anisopliae*. In contrast, Aspergillaceae (*Neosartorya* sp., *A. niger*, and *A. terreus*) and a large number of undefined saprotrophs were associated with CT.

Similarity percentage analysis (SIMPER) also revealed significant fungal compositional differences between Control and other fertilizer groups (N-only and NPK; Figure 7B). At least 27 fungal OTUs responded positively to fertilizer treatments. “NPK” and “N-only” shared several OTUs that were found to be relatively more abundant under those regimes compared to the Control group. Several Penicillium species, *Gibberella fujikuroi*, Cryptococcus, Colletotrichum, *Bionectria ochraleuca* and *Fusarium solani* were well represented in N-only and NPK groups. In the Control group, several mycorrhizal fungal taxa including Glomus and Scutellospora were found to be more abundant than other fertility groups. Furthermore, several plant pathogens including *Aspergillus* species *Aspergillus flavus* and *Neosartorya* sp. *SH8*), *Corynespora cassiicola, Glomerella cingulata*, and *Myrothecium verrucaria* were found to be more abundant in the Control group.

### Response of Plant Pathogenic Fusaria and Oomycetes to Tillage and Fertilizer Treatments

**FIGURE 8:**
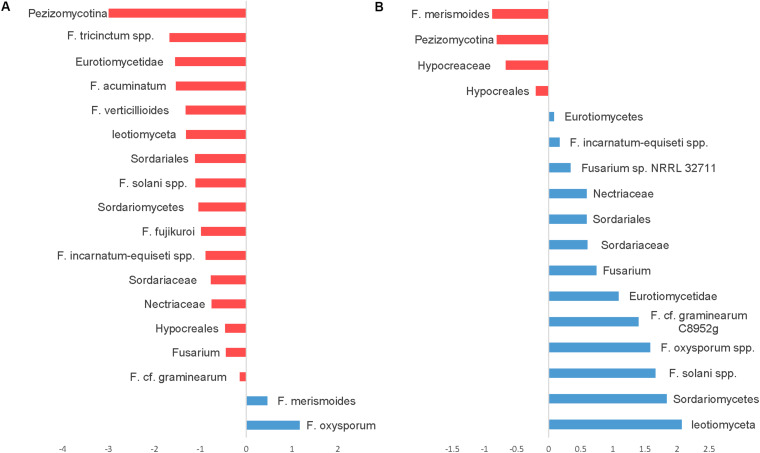
Similarity percentage analysis (SIMPER) of fusaria OTUs affected by tillage and fertilizer treatments. **(A)** Relative abundance of fusaria taxa influenced by tillage treatment. Mean taxa abundances of NT were subtracted from CT. Taxa that are more abundant in NT are right of the center axis (blue), while taxa more abundant in CT (red) are left of center axis. **(B)** Fusaria taxa influenced by fertilizer regimes; NPK and N-only in blue, Control (No fertilizer) in red.

Seedling diseases in soybean and other crops are common and are caused by fungal and oomycetes genera including *Fusaria, Rhizoctonia, Pythium*, and *Phytophthora*. We have identified several OTUs in the fungal ITS dataset classified as Fusarium with very abundant sequences, but because of the conserved nature of ITS sequences among fusaria, fusaria were not assigned at the species level. Nevertheless, we were able to identify at least 26 OTUs at the species level using the translation elongation factor 1 alpha (EF-1α). Notably, using SIMPER analysis OTUs belonging to the *Fusarium solani* species complex, *Fusarium graminearum*, the *Fusarium tricinctum* species complex, *Fusarium acuminatum*, *Fusarium verticillioides*, the *Fusarium incarnatum-equiseti* species complex, and *Fusarium fujikuroi* were relatively more abundant in CT. However, only two OTUs, *Fusarium oxysporum* and *Fusarium merismoides* increased in NT regime compared to CT (Figure 8A).

While the “Fertilizer” factor was not found to significantly impact the overall structure of the detected fusaria communities as shown in the PERMANOVA test (Table 1), few fusaria groups abundances were affected by the fertilizer treatment; the *Fusarium oxysporum* species complex, the *Fusarium solani* species complex, *Fusarium graminearum*, and the *Fusarium incarnatum-equiseti* species complex increased in fertilizer regimes N-only and NPK. Only one *Fusarium* OTU, identified as *F. merismoides*, was found to have a uniform positive response in the Control group (Figure 8B).

Another group of seedlings diseases-causing-pathogens are *Pythium* and *Phytophthora* species. They cause seed rots, damping offs and result in poor emergence. We identified at least 16 OTUs in the *Pythium* genus and 2 OTUs in the Peronosporales family. However, no *Phytophthora* species were detected. *Pythium attrantheridium* was only found in NT plots and absent in CT plots, and *Pythium aff. monospermum* was more abundant under the NT regime. In CT, four OTUs corresponding to *Pythium monospermum*, Pernosporales, *Peronospora manshurica* and *Pythium hypogynum* were found to be more abundant (Supplementary Table S6). The fertilizer effect was not evident on oomycetes communities; none of the oomycete taxa were found to be significantly dissimilar between the fertilizer groups.

## Discussion

The long-term impact of inorganic fertilizers and the adoption of different tillage systems on soil physical factors and nutrient cycling has been extensively studied and described with a wide range of crops and different types of soils worldwide (Alvarez, 2005; Blair et al., 2006; Alvarez and Steinbach, 2009). Tillage and fertilizer regimes have seldom been studied together in term on their effect on microbial populations in the soil, and studies have often been limited to an assessment of the bacterial community structure with fungal and oomycetes communities receiving little to no attention so far (Ceja-Navarro et al., 2010; Ahn et al., 2012; Chaudhry et al., 2012; Aislabie and Deslippe, 2013). In the current study, we aimed to assess the combined effect of select tillage and fertilizer factors in structuring the soil microbial community, including bacteria, fungi, fusaria and oomycetes, especially with respect to affecting the functional diversity and niche-based processes across these taxa. More specifically, we explored induced changes in the soil microbial communities under two tillage regimes (CT and NT) in combination with fertilizers applications (Control, N-only, and NPK) in a long-term corn/soybean rotation field.

This study complements a previous study where soil samples concurrently collected from the same site with the samples used in the current study, were analyzed for physical and chemical properties (Cook and Trlica, 2016). No-till management resulted in an increase in the concentration of organic matter (OM) in the surface soil layer compared with conventional tillage. Despite the higher OM content and nutrient concentrations in the surface layers of soils subjected to NT, in the current study, NT management did not result in an increase in microbial richness nor diversity. This is reflected by the alpha-diversity analysis. In fact, soils under CT seemed to host more diverse bacterial and richer fusaria communities compared to NT soils (Supplementary Table S2), which is in line with similar studies (Smith et al., 2016). Similarly, fungal communities under CT were richer and more diverse than those in NT although the differences were not statistically different under the parameters set for this study. It is to be noted that the only exception was a significantly higher fusaria diversity in NT soils that received NPK fertilization (Supplementary Table S2; Interaction). Several studies have previously reported a correlation between soil compaction and the drop in several microbial parameters such as “number of species,” “microbial enzyme activities” and “microbial catabolic activities” (Pignataro et al., 2012; Janusauskaite et al., 2013). Tilled soils tend to have less organic matter and tend to lose nutrients and soil organic carbon over time (Kaschuk et al., 2010). However, in this study, the increased diversity of bacteria and the prevalence of fusaria in soils subjected to CT may be attributed to the disturbance and mixing of soil and litter caused by tillage, allowing direct contact between bacteria, saprotrophs and the substrate. Tillage redistributes and homogenizes the nutrients and organic matter through the tilled layer, causing a burst in the activity of microbes that are highly competitive, such as saprotrophs (Schmidt et al., 2018). This may explain the more rich and diverse taxonomic profiles often associated with tilled soils. Soil nutrient analysis (Cook and Trlica, 2016) of soils collected from the same long-term study revealed that NT soils had increased amounts of P only in the surface layer (0–10 cm), while P tends to decrease below 10 cm. On the other hand, CT soils had more homogenized P levels throughout the tilled layer. The low P availability to plant roots in NT systems might have promoted the establishment of symbiotic relationships including those with mycorrhizae fungi, especially that these fungi are known to efficiently absorb phosphates from the soil (Kobae, 2019). This is reflected in our data, where a higher prevalence of mycorrhizae fungi was associated with NT (Figure 4A).

In this study, tillage practices resulted in significant shifts in the microbial community structure and composition, and the soil biome responded differently to different tillage regimes. The shifts were evident in bacterial, fungal including fusaria, and oomycetes communities (Table 1 and Figures 1, 2). These observations are in agreement with previous findings (Ceja-Navarro et al., 2010; Oehl et al., 2010; Mathew et al., 2012; Mbuthia et al., 2015). Tillage had its most significant impact on the soil bacterial and fungal communities, followed by Oomycetes and Fusaria communities (Table 1). Results of principal coordinate analysis based on the “tillage variable” underscored separate clustering of tilled and non-tilled soils with a significant degree of variation among the microbial communities associated with those regimes (Figures 1, 2). The long-term tillage treatment emerged as a major driver of fungal trophic modes, possibly due to the formation of distinct habitats for microorganisms under this regime. This is in line with recent publications (Navarro-Noya et al., 2013; Sengupta and Dick, 2015; Degrune et al., 2016). The abundance of fungal symbiotrophs and pathotrophs increased in “NT” soils, whereas fungal total saprotrophs were more abundant in tilled soils “CT” (Figures 3A, 7A). These results support the hypothesis that the absence of soil disturbance, i.e., tillage in our case, favors the growth of fungi forming hyphal networks such as arbuscular mycorrhizal fungi (AMF) (Frey et al., 1999). AMF tend to dominate in no-tillage systems because hyphae are required to contact litters left on the soil surface, which ultimately leads to the enrichment of NT soils in microbial-derived soil organic matter in the form of stable polymers of melanin and chitin (Guggenberger et al., 1999). Several studies have noted that the stable habitat created by conservation tillage selects for beneficial microbial groups that translocate nutrients and resources between the soil and the host plant (Feng et al., 2003; Zhang et al., 2012). In CT fungal saprotrophs seemed to be less susceptible to tillage than mycorrhizal fungi, which might explain the dominance of saprotrophic fungi in tilled soils (Wortmann et al., 2008). Though overall abundance of saprotrophs increased in CT, obligate saprotrophs guilds associated with soil-litter and plant-wood were found relatively more abundant in no-till soils than in tilled soils. The accumulation of crop residues on the surface of no-till soils results in an increase in soil organic matter in the top layer (Govaerts et al., 2009). This may promote the proliferation of obligate saprotrophic fungi. Since conventional tillage leaves less than 15% of crop residue compared to more than 30% in conservation tillage, including no-till, one might expect higher soil organic carbon concentration in no-till soils. However, incorporating the crop residue at 20 cm depth by tillage tends to increase aeration. In addition, tillage brings soil saprotrophs in direct contact with the organic substrate. It has been shown that mycorrhizae fungi can produce a glycoprotein glomalin, which can form a lattice-like coating surrounding soil aggregates, thereby enhancing the stability of soil aggregates, slowing down the decay speed of soil organic carbon, and regulating the activity of soil decomposers (Rillig, 2004; Carvalho et al., 2009; Wu et al., 2015). The buildup of saprotrophs in tilled soils may be explained by soil disturbance initiated by plowing, which can disrupt the hyphal networks of mycorrhizae fungi and destabilize soil aggregates, thereby exposing the organic material to saprobes, speeding the decomposition rate, and accelerating the mineralization of soil organic carbon. Several reports have suggested a link between macro-aggregate turnover and soil carbon dynamics (Jastrow, 1996; Six et al., 2000). In the absence of soil disturbance, inputs of organic debris tend to associate with the mineral matter of soil macro-aggregates. In turn, the soil carbon is rendered physically protected, slowing decomposition and promoting the development of a stable soil structure (Jastrow, 1996; Six et al., 2000). Therefore, in this study, No-till may have promoted soil aggregation processes by developing stable macro-aggregates and thereby physically protecting the soil carbon substrate from microbial access. In contrast, the physical disruption of soil by tilling caused macro-aggregates breakdown and resulted in a higher rate of microbial decomposition leaving the soil vulnerable to erosion and compaction.

Notably, ecological guilds belonging to the “pathotrophic mode” differed in abundance between tillage treatments; the abundance of “mycoparasites,” and “animal pathogens” increased, whereas that of “plant pathogens” declined in no-till soils. The turnover caused by tillage is in line with other studies indicating a positive correlation between the occurrence of AMF and mycoparasites often favored in carbon rich soil (Veach et al., 2018; Pimentel et al., 2020). However, the relatively high abundance of plant pathogens in tilled soils is not consistent with other studies (Steinkellner and Langer, 2004; Degrune et al., 2016).

The effect of tillage was evident also on bacterial communities. The NT regime favored mostly oligotrophic bacteria, e.g., Acidobacteria, Verrucomicrobia, Nitropsirae, and Cyanobacteria characterized by their ability to grow in conditions were resources are limited given their efficiency at scavenging nutrients from recalcitrant OM substrates. While under the CT regime, copiotrophic bacteria that utilize easily decomposable organic material such as Actinobacteria, Thermophilia, Alphaproteobacteria, and Firmicutes, prevailed. The prevalence of fast growing copiotrophic bacteria in CT systems is due to the turnover of the top soil layer, making OM available for these organisms (Powlson et al., 2012). The increased abundance of copiotrophs in tilled soils and oligotrophs in no-till soils have been corroborated in several other studies (Navarro-Noya et al., 2013; Carbonetto et al., 2014; Ramirez-Villanueva et al., 2015). While the majority of soil bacterial communities were shaped according to the oligotrophy-copiotrophy (fast-growing r-strategist vs. slow growing k-strategist) theory, several phyla found in this study to be associated with CT, including Gemmatimonadetes, Firmicutes, and Armatimonadetes, thrive well in low soil moisture, and are known for their tolerance to desiccation and harsh environmental conditions (Zeng et al., 2016).

PICRUSt was applied to the generated 16S rRNA gene data to infer functional traits dominating among tillage treatments. In this study, the functional profiling of the NT soil microbial community revealed abundant representation of genes involved in pathways related to metabolism including “energy metabolism,” “nucleotide metabolism,” “glycan biosynthesis and metabolism,” and “metabolism of cofactors and vitamins” (Figure 5). The enrichment in energy metabolism pathways comprising functions such as carbon fixation in photosynthetic organisms, oxidative phosphorylation; photosynthesis; and methane, nitrogen, and sulfur metabolism, correlates with our taxonomic analysis. That analysis indicates a prevalence of oligotrophic organisms in NT soil involved in photosynthetic activities such as cyanobacteria. In general, functions related to basic metabolism were found more abundant in NT soil. The relatively higher anabolic activity observed in NT soil suggests a larger potential for microbial nitrogen and carbon fixation in those soils in comparison to soils subjected to conventional tillage (Smith et al., 2016). This higher anabolic activity in NT soil may be due to minor soil disturbances characteristic of no-till plots resulting in conditions conducive to enhancing the microbial metabolic rates for major nutrient cycles. In contrast, CT soil showed a higher abundance of genes involved in secondary metabolism (amino acid metabolism, lipid metabolism), degradation of complex compounds and soil pollutants (xenobiotics biodegradation and metabolism, metabolism of terpenoids and polyketides) and environmental adaptation. The pathways observed in CT soil are characteristics of a fitness response to environmental stress. For instance, the higher abundance of lipid metabolism results in increased fatty acids production which are important components of microbial membranes and are crucial for microbes survival in stress environments (Fozo and Quivey, 2004). Furthermore, the defense mechanisms pathways (i.e., xenobiotics biodegradation and metabolism, metabolism of terpenoids and polyketides, cell growth and death, and environmental adaptation) enriched in CT indicate an accumulation of toxic compounds, and an elevated microbial competition in CT soil, thereby selecting for increased antibiotic production and resistance.

By contrast to tillage, in this study, fertilizer treatments impacted only fungal and bacterial communities’ composition, with oomycetes and fusaria communities not significantly impacted (Table 1). While taxonomic characterization revealed community pattern changes between different fertilizer treatments, the functional analysis of the data suggests that the influence exerted on functional traits by the tested fertilizer treatments is limited. The data reflected weak shifts in functional diversity using FUNGuild inference, with only fungal symbiotrophs dominating in the Control as is expected with a non-amended soil (Figure 3B). Similarly, in bacteria, PICRUSt inferred no functional variation among the different fertilizer treatments. Discrepancies between taxonomic characterization and PICRUSt functional inference suggests potential functional redundancy among the different community structures associated with each fertilizer treatment.

Fusarium species are important plant pathogens and are ubiquitous in soils as colonizers of living plants or debris. They are capable of surviving as conidia, chlamydospores, and as mycelia associated with plant debris for extended periods of time (Steinkellner and Langer, 2004). Tillage practices are often advocated as tools to manage. Nevertheless, the effect of tillage to reduce the incidence of *Fusarium* species has been controversial (David Miller et al., 1998; Steinkellner and Langer, 2004; Hofgaard et al., 2016). In this study, *Fusarium* species richness and diversity were found to increase in CT regime. A large number of *Fusarium* species dominated in CT, whereas only *F. oxysporum* and *F. merismeroides* were more prevalent in the NT regime. While our results align with more recent findings by Weber et al. (2001) and Joseph et al. (2016), they seem to contradict findings by Bailey (1996); Dill-Macky and Jones (2000), and others. It is important to note here that the majority of these studies focused on a single pathogenic *Fusarium* specie, often associated with a specific disease. In contrast, this study covers the frequency of the whole fusarium community under different tillage treatments. Our findings suggest that conventional tillage practices, under similar soil and climatic conditions, would not reduce the frequency and abundance of *Fusarium* species. In this study, NT regime seemed to be supporting a selective and balanced population of microbial species that are natural enemies of soil-borne pathogens in the upper layers of soil. Krupinsky et al. (2002) and Perez-Brandán et al. (2012) reported similar findings where zero-tillage resulted in beneficial effects on soil quality and enhanced natural suppression of fungal pathogens.

To date, to our knowledge, there exists no assessment of the role of tillage practices and fertilizer inputs on oomycetes community structure and composition. This study is a first in addressing this question. In this study, tillage significantly affected the oomycetes community structure, whereas different fertilizer inputs did not result in significant modifications in that structure. In CT managed soil, more oomycetes taxa prevailed in relative abundance than in NT. Notably, the Peronosporales and *Peronospora manshurica* dominated in NT; the latter is known to cause downy mildew on soybean and is prevalent in Illinois (Lim, 1978). Conversely, *P. attrantheridium* was relatively more abundant in the NT soils. *P. attrantheridium* is a known pathogen of both corn and soybean, which might have led to a buildup of its population in the NT soil due to the accumulation of crop residues (Broders et al., 2007).

In summary, this study shows that different management practices using no-till or conventional till with and without fertilizer additions altered soil microbial structure and community functional diversity. We found that tillage disturbance asserted the largest influence on microbial ecology in comparison to fertilizer additions. Community composition and function varied substantially between tilled and non-tilled plots, in contrast there was limited differences in response to different fertility treatments. In our study, no-till management shifted microbial communities toward a symbiotic relationship including mycorrhizae fungi, mycoparasites, nematophagous fungi, and other oligotrophs that efficiently absorb nutrients from the soil and protect the host plant. In addition to saprotrophs and contrary to our hypothesis, diversity and abundance of plant pathogenic fungi increased in conventional tilled plots. Similarly, fast growing competitors including saprotrophs and copiotrophs organisms were favored in tilled plots due to the breakdown of macro-aggregates caused by soil disturbance.

Within the local agronomic and pedologic context of Southern Illinois, tillage impacted resilient soil microbial communities, favoring opportunistic commensal microbes rather than beneficial mutualistic organisms. This apparent niche turnover may have a long-term detrimental impact on soil health while also making the soil more vulnerable to erosion. No-till when combined with NPK management appeared to promote niche-based processes that support high crop yield while reducing nutrient losses and soil erosion. In order to improve agricultural management practices, there is a need to better understand the relationship between these practices and changes in microbial community structure and lifestyles. Data presented here indicates that cultural practices can be exploited as tools to promote sustainable agricultural production while maintaining high yield by managing the soil microbiome structure and function.

## Data Availability Statement

The datasets generated for this study can be found in the https://www.ncbi.nlm.nih.gov/sra/SRP242303.

## Author Contributions

AYS and AF conceived and designed the experiments. RC contributed to management of the field site. AYS and HA contributed the sampling, DNA extraction, and amplicon sequencing. AYS and AS analyzed the data. AYS and MP wrote the manuscript. All the authors reviewed and approved the manuscript.

## Conflict of Interest

The authors declare that the research was conducted in the absence of any commercial or financial relationships that could be construed as a potential conflict of interest.

## Footnotes

1 https://github.com/UMNFuN/FUNGuild

2 https://github.com/cran/randomForest

## Supplementary Material

FIGURE S1 OTU rarefaction curves. **(A)** Bacterial, **(B)** Fungal, **(C)** Oomycetes, and **(D)** Fusaria. Barcodes sequences on the right represent each sample.

FIGURE S2 Random forest importance plot of top bacterial OTUs ranked based on the mean decrease accuracy for each CT and NT regimes. **(A)** At the phylum rank, and **(B)** at the class rank; CT, conventional till in red, and NT, no till in green.

TABLE S1 Tillage and fertilizer treatments used in the current study (1970–2015). Fertilizer treatments were only applied in corn years.

TABLE S2 Effect of long-term tillage and fertilizer treatments on microbial alpha diversity; OTUs observed and Shannon (H) indices were calculated for each microbial community group in response to treatment.

TABLE S3 Similarity percentage analysis of bacteria at the lowest OTU level between CT and NT.

TABLE S4 Similarity percentage analysis of bacteria at the lowest OTU level between Control and N-only fertilizer groups.

TABLE S5 Similarity percentage analysis of bacteria at the lowest OTU level between Control and NPK fertilizer groups.

TABLE S6 Similarity percentage analysis of oomycetes at the lowest OTU level between CT and NT tillage groups.

DATA SHEET S1 Detailed Fluidigm access array PCR amplification protocol.
