# Supplementary figures and images for: Microbial Communities Associated With Long-Term Tillage and Fertility Treatments in a Corn-Soybean Cropping System
Source: Front Microbiol. 2020 Jun 25;11:1363. doi: 10.3389/fmicb.2020.01363 (PMC7330075; doi:10.3389/fmicb.2020.01363)

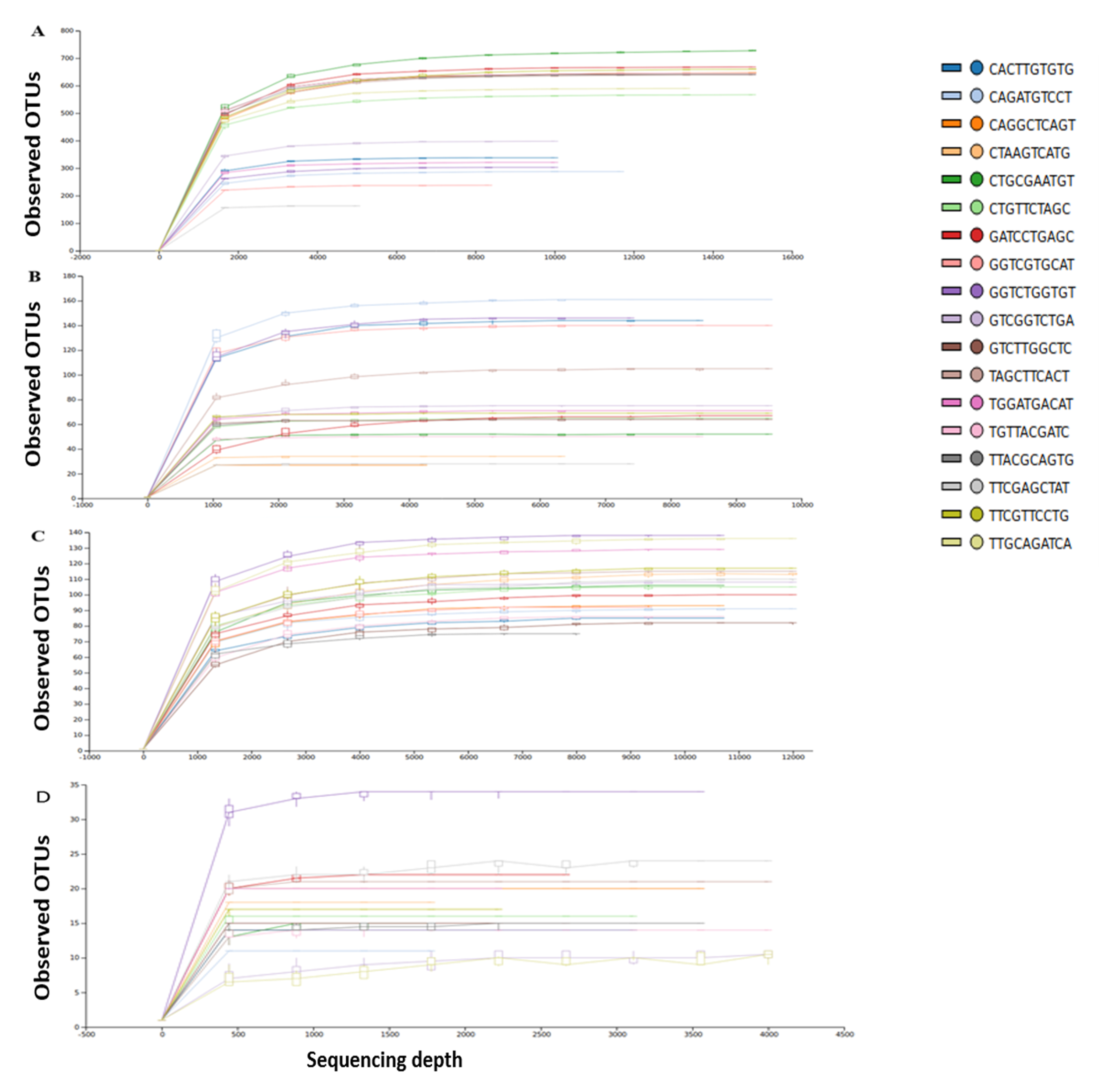

Supplement: FIGURE S1 — OTU rarefaction curves. (A) Bacterial, (B) Fungal, (C) Oomycetes, and (D) Fusaria. Barcodes sequences on the right represent each sample. [file Image_1.TIF]

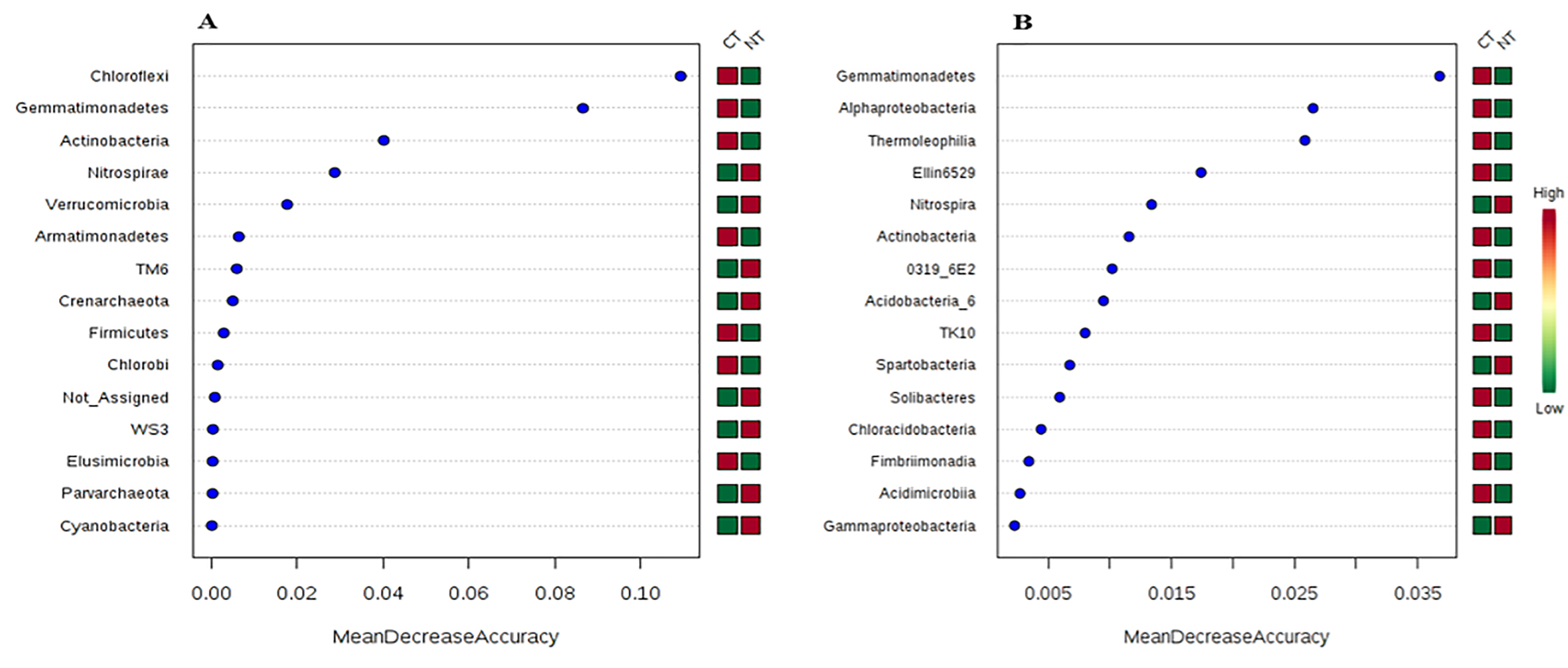

Supplement: FIGURE S2 — Random forest importance plot of top bacterial OTUs ranked based on the mean decrease accuracy for each CT and NT regimes. (A) At the phylum rank, and (B) at the class rank; CT, conventional till in red, and NT, no till in green. [file Image_2.tif]
