# Supplementary material for: Microbial Communities Associated With Long-Term Tillage and Fertility Treatments in a Corn-Soybean Cropping System
Source: Front Microbiol. 2020 Jun 25;11:1363. doi: 10.3389/fmicb.2020.01363 (PMC7330075; doi:10.3389/fmicb.2020.01363)
Supplement: TABLE S1 — Tillage and fertilizer treatments used in the current study (1970–2015). Fertilizer treatments were only applied in corn years. [file Table_1.DOCX]

**Table S1** Tillage and fertilizer treatments used in the study (1970 to 2015). Fertilizer treatments were only applied in corn years.

| **Tillage treatment** | |  | **Fertilizer treatment** | N-P-K kg ha-^1^ | | |
| --- | --- | --- | --- | --- | --- | --- |
|  |  |  |  | 1970-1973 | 1974-1999 | 2000-2015 |
|  | Seedbed preparation steps |  | Control | 0-0-0 | 0-0-0 | 0-0-0 |
| Conventional (CT) | Moldboard plow, tandem disk, cultivator, culti-mulcher |  | N-only | 140-0-0 | 196-0-0 | 196-0-0 |
| No-till (NT) | none |  | NPK | 140-25-116 | 196-39-168 | 196-24-140 |
