# Supplementary material for: Microbial Communities Associated With Long-Term Tillage and Fertility Treatments in a Corn-Soybean Cropping System
Source: Front Microbiol. 2020 Jun 25;11:1363. doi: 10.3389/fmicb.2020.01363 (PMC7330075; doi:10.3389/fmicb.2020.01363)
Supplement: TABLE S2 — Effect of long-term tillage and fertilizer treatments on microbial alpha diversity; OTUs observed and Shannon (H) indices were calculated for each microbial community group in response to treatment. [file Table_2.DOCX]

|  | Bacteria | | | | Fungi | |  | Fusaria | | |  | Oomycetes | |
| --- | --- | --- | --- | --- | --- | --- | --- | --- | --- | --- | --- | --- | --- |
| Treatment* | H |  | OTU |  | H | OTU |  | H |  | OTU |  | H | OTU |
|  | Tillage | | | | | | | | | | | | |
| CT | 8.22 | a** | 508.56 |  | 4.33 | 81.44 |  | 3.51 |  | 21.27 | a | 3.57 | 97.44 |
| NT | 7.40 | b | 435.44 |  | 4.28 | 73.71 |  | 2.68 |  | 13.44 | b | 3.47 | 111.00 |
|  | Fertilizer | | | | | | | | | | | | |
| Control | 7.84 |  | 472.67 |  | 4.04 | 51.61 |  | 2.50 | b | 14.40 |  | 3.69 | 110.50 |
| N | 8.16 |  | 505.67 |  | 4.56 | 85.95 |  | 3.34 | a | 19.50 |  | 3.59 | 106.50 |
| NPK | 7.43 |  | 437.67 |  | 4.32 | 95.17 |  | 3.44 | a | 18.17 |  | 3.27 | 95.67 |
|  | Interaction | | | | | | | | | | | | |
| CT-Control | 8.09 |  | 485.67 |  | 3.68 | 60.67 |  | 3.12 | AB | 18.81 | AB | 3.35 | 101.33 |
| CT-N | 8.40 |  | 536.33 |  | 4.44 | 89.33 |  | 3.90 | A | 24.67 | A | 3.62 | 107.00 |
| CT-NPK | 8.17 |  | 503.67 |  | 4.87 | 94.33 |  | 3.51 | A | 20.33 | AB | 3.73 | 84.00 |
| NT-Control | 7.59 |  | 459.67 |  | 4.40 | 42.56 |  | 2.78 | B | 10.00 | B | 4.03 | 119.67 |
| NT-N | 7.91 |  | 475.00 |  | 4.67 | 82.56 |  | 2.78 | AB | 14.33 | AB | 3.56 | 106.00 |
| NT-NPK | 6.69 |  | 371.67 |  | 3.77 | 96.00 |  | 3.37 | A | 16.00 | AB | 2.82 | 107.33 |
|  |  |  |  |  |  |  |  |  |  |  |  |  |  |
|  | P values | | | | | | | | | | | | |
| Tillage | 0.037 |  | 0.449 |  | 0.750 | 0.941 |  | 0.053 |  | 0.026 |  | 0.171 | 0.773 |
| Fertilizer | 0.281 |  | 0.844 |  | 0.309 | 0.768 |  | 0.001 |  | 0.180 |  | 0.197 | 0.425 |
| TillagexFertilizer | 0.458 |  | 0.888 |  | 0.938 | 0.441 |  | 0.010 |  | 0.442 |  | 0.300 | 0.097 |

**Table S2** Effect of long-term tillage and fertility treatments on microbial alpha diversity; OTU observed and Shannon (H) indices were calculated for each microbial community group in response to treatment

*Abbreviations: CT= conventional tillage, NT= no tillage, N= nitrogen, NPK= nitrogen, phosphorous and potassium. **Treatments within each effect followed by different letters are significantly different at *P* ≤ 0.05. Means with no letters assigned are not significantly different.
