# Supplementary material for: Microbial Communities Associated With Long-Term Tillage and Fertility Treatments in a Corn-Soybean Cropping System
Source: Front Microbiol. 2020 Jun 25;11:1363. doi: 10.3389/fmicb.2020.01363 (PMC7330075; doi:10.3389/fmicb.2020.01363)
Supplement: DATA SHEET S1 — Detailed Fluidigm access array PCR amplification protocol. [file Data_Sheet_1.DOCX]

**Fluidigm Access Array Amplification**

Prior to amplification all DNA samples were measured on a Qubit (Life Technologies) using the High Sensitivity DNA Kit. Samples were diluted to 2 ng/ul concentrations. A mastermix for amplification was prepared using the Roche High Fidelity Fast Start Kit and 20x Access Array loading reagent according to Fluidigm protocols. For each sample the following reagents were combined

0.5 ul -10X FastStart Reaction Buffer without MgCl2

0.9 ul -25 mM MgCl2

0.25 ul – DMSO

0.1 ul -10 mM PCR grade Nucleotide Mix

0.05 ul -5 U/ul FastStart High Fidelity Enzyme Blend

0.25 ul – 20X Access Array Loading Reagent

0.95 ul – Water

Mastermix was aliquoted to 48 wells of a PCR plate. To each well, 1 ul DNA sample and 1 ul Fluidigm Illumina linkers with unique barcode were added. In a separate plate, primer pairs were prepared and aliquoted. 20X primer solutions were prepared by adding 2 ul of each forward and reverse primer (50uM), 5 ul of 20X Access Array Loading Reagent and water to a final volume of 100 ul. Final primer concentration in the reactions were 50 nM each.

Primer sequences for respective variable regions are listed below:

| Primer target | Primer name | Locus-specific primer sequence |
| --- | --- | --- |
| ITS3_ITS4 | ITS3 | 5'-GCATCGATGAAGAACGCAGC |
|  | ITS4 | 5'-TCCTCCGCTTATTGATATGC |
|  |  |  |
| V4_515F_806R_New | V4_515F_New | 5'-GTGYCAGCMGCCGCGGTAA |
|  | V4_806R_New | 5'-GGACTACNVGGGTWTCTAAT |
|  |  |  |
| EF1aF_EF1aR | EF1aF | 5'-TCGTCATCGGCCACGTCGACTC |
|  | EF1aR | 5'-CCTTACCGAGCTCRGCGGCTT |
|  |  |  |
| ITS6_ITS7 | ITS6 | 5'-GAAGGTGAAGTCGTAACAAGG |
|  | ITS7 | 5'-AGCGTTCTTCATCGATGTGC |

4 ul of sample was loaded in the sample inlets and 4 ul of primer loaded in primer inlets of a previously primed Fluidigm 48.48 Access Array IFC. The IFC was placed in an AX controller (Fluidigm Corp.) for microfluidic loading of all primer/sample combinations. Following the loading stage, the IFC plate was loaded on the Fluidigm Biomark HD PCR machine and samples were amplified using the following Access Array cycling program without imaging:

**PCR Stages Number of Cycles**

50ºC 2 minutes 1

70ºC 20 minutes 1

95ºC 10 minutes 1

95ºC 15 seconds

55ºC 30 seconds

72ºC 1 minute 10

95ºC 15 seconds

80ºC 30 seconds

60ºC 30 seconds

72ºC 1 minute 2

95ºC 15 seconds

55ºC 30 seconds

72º 1 minute 8

95ºC 15 seconds

80ºC 30 seconds

60ºC 30 seconds

72ºC 1 minute 2

95ºC 15 seconds

55ºC 30 seconds

72ºC 1 minute 8

95ºC 15 seconds

80ºC 30 seconds

60ºC 30 seconds

72ºC 1 minute 5

Following amplification 2ul of Fluidigm Harvest Buffer was loaded in the sample inlets and loaded on the AX controller for harvesting PCR products. Harvested product was then transferred to a new 96 well plate quantified on a Qubit fluorimeter and stored at -20C. All samples were run on a Fragment Analyzer (Advanced Analytics, Ames, IA) and amplicon regions and expected sizes confirmed. Samples were then pooled in equal amounts according to product concentration. The pooled products were then size selected on a 2% agarose E-gel (Life Technologies) and extracted from the isolated gel slice with Qiagen gel extraction kit (Qiagen). Cleaned size selected products were run on an Agilent Bioanalyzer to confirm appropriate profile and determination of average size.

**Fluidigm Access Array Second Stage Protocol**

Harvested product was then transferred to a new 96 well plate and diluted 1:100 in water. 1 ul of diluted product was used for a second round of amplification with Illumina linkers and barcodes.

For second round PCR the following reagents were combined:

2.0 ul -10X FastStart Reaction Buffer without MgCl2

3.6 ul -25 mM MgCl2

1.0 ul – DMSO

0.4 ul -10 mM PCR grade Nucleotide Mix

0.2 ul -5 U/ul FastStart High Fidelity Enzyme Blend

7.8 ul – Water

15 ul of reagent mix was combined with 1 ul diluted first round PCR product and 4 ul of Illumina linker barcodes.

The PCR cycling program consisted of

95C, 10 min. 1 cycle

95C 15sec.

60C 30 sec.

72C 1min. 15cycles

72C 3 min. extension

Products were quantified on a Qubit fluorimeter and stored at -20C. All samples were run on a Fragment Analyzer (Advanced Analytics, Ames, IA) and amplicon regions and expected sizes confirmed. Samples were then pooled in equal amounts according to product concentration. The pooled products were then size selected on a 2% agarose E-gel (Life Technologies) and extracted from the isolated gel slice with Qiagen gel extraction kit (Qiagen). Cleaned size selected products were run on an Agilent Bioanalyzer to confirm appropriate profile and determination of average size.
